# Lack of association of genetic variants in genes of the endocannabinoid system with anorexia nervosa

**DOI:** 10.1186/1753-2000-2-33

**Published:** 2008-11-17

**Authors:** Timo Dirk Müller, Kathrin Reichwald, Günter Brönner, Jeanette Kirschner, Thuy Trang Nguyen, André Scherag, Wolfgang Herzog, Beate Herpertz-Dahlmann, Peter Lichtner, Thomas Meitinger, Matthias Platzer, Helmut Schäfer, Johannes Hebebrand, Anke Hinney

**Affiliations:** 1 Department of Child and Adolescent Psychiatry, University of Duisburg-Essen, Essen, Germany; 2 Leibniz Institute for Age Research – Fritz Lipmann Institute (FLI), Jena, Germany; 3 Biocenter of the University of Wuerzburg, Wuerzburg, Germany; 4 Institute of Medical Biometry and Epidemiology, Philipps-University, Marburg, Germany; 5 Institute of Medical Informatics, Biometry and Epidemiology, University of Duisburg-Essen, Essen, Germany; 6 Klinik für Psychosomatische und Allgemeine Klinische Medizin, Universitätsklinikum Heidelberg, University of Heidelberg, Germany; 7 Department of Child and Adolescent Psychiatry and Psychotherapy, University Clinics, Technical University of Aachen, Aachen, Germany; 8 Institute of Human Genetics, Technical University Munich, Munich, Germany; 9 GSF – National Research Center for Environment and Health, München-Neuherberg, Germany; 10 Department of Psychiatry, University of Cincinnati Genome Research Institute, Cincinnati, OH, USA

## Abstract

**Background:**

Several lines of evidence indicate that the central cannabinoid receptor 1 (CNR1) as well as the major endocannabinoid degrading enzymes fatty acid amide hydrolase (FAAH), N-acylethanolamine-hydrolyzing acid amidase (NAAA) and monoglyceride lipase (MGLL) are implicated in mediating the orexigenic effects of cannabinoids. The aim of this study was to analyse whether nucleotide sequence variations in the *CNR1*, *FAAH*, *NAAA *and *MGLL *genes are associated with anorexia nervosa (AN).

**Methods:**

We analysed the association of a previously described (AAT)n repeat in the 3' flanking region of CNR1 as well as a total of 15 single nucleotide polymorphisms (SNPs) representative of regions with restricted haplotype diversity in *CNR1*, *FAAH*, *NAAA *or *MGLL *in up to 91 German AN trios (patient with AN and both biological parents) using the transmission-disequilibrium-test (TDT). One SNP was additionally analysed in an independent case-control study comprising 113 patients with AN and 178 normal weight controls. Genotyping was performed using matrix-assisted laser desorption/ionization time-of-flight mass spectrometry, ARMS-PCR or using 3730xl capillary sequencers.

**Results:**

The TDT revealed no evidence for association for any of the SNPs or the (AAT)n repeat with AN (all two-sided uncorrected p-values > 0.05). The lowest p-value of 0.11 was detected for the A-allele of the *CNR1 *SNP rs1049353 for which the transmission rate was 59% (95% confidence interval 47%...70%). Further genotyping of rs1049353 in 113 additional independent patients with AN and 178 normal weight controls could not substantiate the initial trend for association (p = 1.00).

**Conclusion:**

As we found no evidence for an association of genetic variation in *CNR1*, *FAAH, NAAA and MGLL *with AN, we conclude that genetic variations in these genes do not play a major role in the etiology of AN in our study groups.

## Background

Anorexia nervosa (AN) is an eating disorder with unknown etiology. The multifactorial pathogenesis of AN has been emphasized in various studies [1-3]. Accordingly, heritability estimates derived from twin studies revealed that 58–76% of the variance of AN can be explained by genetic factors [4]. The highest incidence for the development of AN is around puberty and patients with AN are typically characterized by an abnormal eating behaviour with disturbances of attitudes towards body weight and shape [5]. Therefore, it is reasonable that genetic factors regulating food intake and body weight are implicated in the pathogenesis of AN [1,2,6,7].

One of the endogenous systems that, due to its therapeutic potential in the treatment of obesity, recently reached scientific interest is the endocannabinoid system. Both exogenous (e.g. Δ^9^-tetrahydrocannabinol) and endogenous cannabinoids (e.g. anandamide and 2-arachidonoylglycerol (2-AG)) stimulate food intake through activation of the cannabinoid receptor 1 (CNR1) [8]. In contrast, inhibition of CNR1 signalling through administration of the selective inverse agonist rimonabant (Acomplia^®^) decreases food intake in both rodents [9-11] and humans [12,13]. The endocannabinoid system further interacts with the leptinergic system; obese rodents with disturbed leptin signal transduction (*ob/ob *and *db/db *mice as well as *fa/fa *rats) show elevated levels of anandamide and 2-AG in the hypothalamus. Vice versa, leptin treatment of *ob/ob *mice decreased hypothalamic levels of both, anandamide and 2-AG [11]. Accordingly, compared to age matched normal weight controls, serum levels of anandamide are increased in patients with AN. Additionally, plasma levels of leptin are negatively correlated with anandamide in both, patients with AN and normal weight healthy controls [2]. In light of these observations, it has previously been suggested that the endocannabinoid system might be implicated in the etiology of AN, in particular through its interaction with the leptinergic system [2,7].

Hypoleptinemia is a cardinal feature of prolonged semi-starvation that entails various metabolic and neuroendocrine alterations which are typically observed in patients with acute AN [5,14] The most prominent neuroendocrine alterations mediated by semi-starvation induced hypoleptinemia include amenorrhea, osteopenia/osteoporosis, and alterations of the hypothalamic-pituitary-gonadal (HPG) and -adrenal (HPA) axis [5,14]. Additionally, several lines of evidence indicate that hypoleptinemia entails development of hyperactivity in patients with AN [5,14-18]. However, the implication of the endocannabinoid system in body weight regulation together with its interaction with the leptinergic system makes it a plausible system implicated in the pathogenesis of AN [2,7].

The most prominent endocannabinoids are N-arachidonoylethanolamine (anandamide) [19] and 2-arachidonoylglycerol (2-AG) [20]. Both are synthesised through cells on demand and undergo a rapid degradation through specific hydrolases and lipases [8,21,22]. The most prominent endocannabinoid degrading enzymes are the fatty acid amide hydrolase (FAAH), the N-acylethanolamine-hydrolyzing acid amidase (NAAA) and the monoglyceride lipase (MGLL) [23]. FAAH is a membrane-bound 60–65 kDa protein that is widely distributed throughout the brain and the periphery [23,24]. Under alkaline conditions, FAAH rapidly inhibits the orexigenic effects of anandamide by degrading it to ethanolamine and arachidonic acid [25,26]. NAAA is an enzyme with similar function but has, in contrast to FAAH, its pH optimum at 4.5–5 [27,28]. The monoglyceride lipase (MGLL) is a serine hydrolase that hydrolyses 2-AG in glycerol and arachidonic acid [22]. As endocannabinoids stimulate food intake through activation of CNR1 and as FAAH, NAAA and MGLL counteract the orexigenic effects of endocannabinoids through their rapid degradation, genetic variation of *CNR1 *leading to decreased receptor signalling as well as genetic variation of *FAAH, NAAA and MGLL *leading to increased enzyme activity might be implicated in the etiology of AN.

We performed association studies to analyse whether allelic variation in *CNR1*, *FAAH*, *NAAA *and *MGLL *is related to the AN phenotype. We therefore genotyped the previously described (AAT)_n _repeat in the 3' flanking region of *CNR1 *as well as a total of 15 SNPs representative of regions with restricted haplotype diversity in *CNR1 *(rs2180619, rs806379, rs1535255, rs2023239 and rs1049353), *FAAH *(rs932816, rs324420, rs324419, rs2295632 and rs873978), *NAAA *(rs2292534, rs6532046, rs10518142 and rs874546) and *MGLL *(rs893294) in up to 91 German AN trios (patient with AN and both biological parents). Genotyping was performed using matrix-assisted laser desorption/ionization time-of-flight mass spectrometry or allele specific polymerase chain reaction (ARMS-PCR). The AAT_14 _repeat allele of *CNR1 *has recently been found to be associated with the binge eating/purging type of AN whereby the AAT_13 _repeat allele tended to be preferentially transmitted to patients with the restricting type of AN [29]. Furthermore, SNP rs324420 (c.385C/A) in the *FAAH *gene, leading to decreased enzyme activity and thus increased levels of endocannabinoids and presumably increased food intake, was recently found to be associated with obesity [30] and drug abuse [31]. The *CNR1 *haplotype comprising the minor alleles of SNP rs806379 (T-allele), rs1535255 (G-allele) and rs2023239 (C-allele) has further been shown to be associated with drug and alcohol abuse in European and African Americans [32].

## Methods

The ascertainment strategy was previously described in detail [33]. Written informed consent was given by all participants and in the case of minors, by their parents. The study was approved by the ethics committees of the Universities of Marburg and Duisburg-Essen and carried out according to the Declaration of Helsinki.

*Study group 1 (AN trios) *comprised 91 (3 male) patients with AN (mean age 15.72 ± 2.04 years, mean BMI 15.42 ± 2.39 kg/m^2^) and both biological parents (mean age 46.73 ± 5.67, mean BMI 26.22 ± 4.16 kg/m^2^).

*Study group 2 (cases and controls) *included 204 patients with AN (113 patients with AN independent from study group 1). The 113 (7 male) independent patients included 65 (3 male) individuals with acute AN and 48 (4 male) individuals from a catamnestic study with a history of AN. The acute patients had a mean age of 22.47 ± 11.67 years and a mean BMI of 14.81 ± 2.29 kg/m^2^. The catamnestic individuals had a mean age of 33.60 ± 7.22 years and a mean BMI of 20.78 ± 2.05 kg/m^2^. In total, the 204 (10 male) patients had a mean age of 22.13 ± 10.03 years, a mean BMI of 16.46 ± 3.30 kg/m^2^. The control group comprised 178 healthy normal weight individuals with a mean age of 24.58 ± 2.56 years and a mean BMI of 21.76 ± 1.08 kg/m^2^. All patients with AN fulfilled the diagnostic criteria for AN according to the diagnostic and statistical manual of mental disorders (DSM-IV) [34].

Genotyping was performed using matrix-assisted laser desorption/ionization time-of-flight mass spectrometry (MALDI-TOF MS, Sequenom, San Diego, CA). Only the *CNR1 *SNP rs1049353 was genotyped using ARMS-PCR as described previously [35]. The *CNR1 *(AAT) trinucleotide repeat was genotyped using 3730xl capillary sequencers (Applied Biosystems) and GeneMapper software (Version 4.0, Applied Biosystems). Primers for analyses of *CNR1 *variations were derived from genomic entry AL136096.7; rs1049353 F_out_: 5'-GGA CTC GGA CTG CCT GCA CAA A-3'; R_out_: 5'-AAA TTC TTT TCC TGT GCT GCC AGG G-3', F_in_: 5'-CAG AAA GCT GCA TCA AGA GCC CG-3', R_in_: 5'-GAC ATG GTT ACC TTG GCA ATC TTG CCT-3' (product size outer primers: 175 bp, G-allele: 120 bp, A-allele: 105 bp); (AAT) trinucleotide repeat: F (FAM-labelled): 5'-CCT TCT CCC AGC ACA ATC AT-3', R: 5'-TAC ATC TCG GTG TGT GAT GTT CC A TGT TCC-3' (PCR-product size based on genomic entry AL136096.7: 277 bp). SNP assays for analyses with MALDI-TOF mass spectrometry were designed with the SpectroDesigner software (Sequenom). For validity of genotypes, alleles were rated independently by at least two experienced individuals. Discrepancies were resolved unambiguously either by reaching consensus or by retyping.

The family-based association analyses were done applying transmission disequilibrium tests (TDT) [36] while the software FAMHAP (v16; ) was used to investigate the haplotypes consisting of the genotyped SNPs within the respective genes. Power considerations for the A-allele of rs1049353 were performed using the program Quanto (v 1.2.3; ) at the one-sided significance level of 0.05. Here, we assumed an AN prevalence of 0.5% and an allele frequency of 0.26 (accoding to , CEU sample). Exact Cochran-Armitage trend test, implemented in SAS, was applied to test for association of the *CNR1 *SNP rs1049353 in case-control data. If not indicated otherwise, all reported p-values are two-sided and were not corrected for multiple testing as none of the null hypotheses was rejected. A significance level of α = 0.05 (two-sided) was applied.

## Results

The Transmission-Disequilibrium Test (TDT) revealed no indication for an association of the analysed SNPs or the (AAT)_n _repeat with AN (Table 1). For the A-allele of the *CNR1 *SNP rs1049353 the lowest p-value was 0.11 for an estimated transmission rate of 59% (95% confidence interval 47%...70%). Applying conditional logistic regression on the trio data, we obtained a multiplicative OR of the A-allele at 1.43 (95% confidence interval 0.91...2.25). Hence, in assuming a true effect of such size the power of our consecutive case-control study comprising 113 additional independent patients with AN and 178 normal weight controls would be about 60%. The initial finding, however, could not be substantiated by the case-control approach (exact p = 1.00). Similarly, a combined case-control analysis including the 91 patients from the family-trios (204 patients with AN and 178 controls) revealed no association of the A-allele of rs1049353 with the AN phenotype (exact p = 0.27; multiplicative OR 1.20; 95% CI 0.88...1.65).

**Table 1:** Genotypes and TDT results of the analysed variants in *CNR1*. *NAAA *and *MGLL *in the AN trios

**Gene**	**SNP^1,2^**	**Alleles^3 ^major/minor**	**Location Exchange**	**N^4^**	**Genotypes (%)^5^**	**Allele frequ.^6^**	**Transm. rate^7^**	**p-value^8^**
					A/A 26 (42.62)	A: 0.64		
					A/G 26 (42.62)			
*CNR1*	rs2180619	-22,959A/G	Putative promoter	61	G/G 9 (14.75)	G: 0.36	0.49 (G)	1.00

					A/A 16 (26.23)	A: 0.55		
					A/T 35 (57.38)			
*CNR1*	rs806379	-6,274A/T	Intron 2	61	T/T 10 (16.39)	T: 0.45	0.52 (T)	0.90

					T/T 43 (70.49)	T: 0.84		
					T/G 16 (26.23)			
*CNR1*	rs1535255	-6,215T/G	Intron 2	61	G/G 2 (3.28)	G: 0.16	0.50 (G)	1.00

					T/T 43 (70.49)	T: 0.84		
					T/C 16 (26.23)			
*CNR1*	rs2023239	-5,489T/C	Exon 3 Non-coding	61	C/C 2 (3.28)	C: 0.16	0.50 (C)	1.00

					G/G 38 (41.76)	G: 0.65		
					G/A 43 (47.25)			
*CNR1*	rs1049353	1,359G/A	Exon 4 Thr453Thr	91	A/A 10 (10.99)	A: 0.35	0.59 (A)	0.11

					G/G 33 (54.10)	G: 0.75		
					G/A 26 (42.62)			
*FAAH*	rs932816	-272G/A	Putative promoter	61	A/A 2 (3.28)	A: 0.25	0.39 (A)	0.13

					C/C 40 (66.67)	C: 0.80		
					C/A 16 (26.67)			
*FAAH*	rs324420	10,741C/A	Exon 3 Thr129Pro	60	A/A 4 (6.67)	A: 0.20	0.56 (A)	0.61

					G/G 49 (80.33)	G: 0.89		
					G/A 11 (18.03)			
*FAAH*	rs324419	11,966G/A	Exon 7 Cys299Cys	61	A/A 1 (1.64)	A: 0.11	0.59 (A)	0.52

					G/G 60 (98.36)	G: 0.99		
					G/A 1 (1.64)			
*FAAH*	rs873978	13,883G/A	Intron 7	61	A/A 0 (0.00)	A: 0.01	1.00 (A)	1.00

					C/C 34 (56.67)	C: 0.73		
					C/A 20 (33.33)			
*FAAH*	rs2295632	19,542C/A	3'UTR	60	A/A 6 (10.0)	A: 0.27	0.42 (A)	0.42

					G/G 34 (56.67)	G: 0.75		
					G/A 22 (36.67)			
*NAAA*	rs2292534	368A/G	Intron 1	60	A/A 4 (6.67)	A: 0.25	0.57 (A)	0.42

					A/A 38 (62.30)	A: 0.78		
					A/T 19 (31.15)			
*NAAA*	rs4859567	9,263A/T	Intron 3	61	T/T 4 (6.56)	T: 0.22	0.43 (T)	0.42

					G/G 36 (60.00)	G: 0.78		
					G/T 21 (35.00)			
*NAAA*	rs10518142	19,229G/T	Intron 5	60	T/T 3 (5.00)	T: 0.22	0.63 (T)	0.13

					C/C 22 (36.07)	C: 0.61		
					C/T 31 (50.82)			
*NAAA*	rs6819442	22,995C/T	Intron 9	61	T/T 8 (13.11)	T: 0.39	0.56 (T)	0.42

					T/T 36 (59.02)	T: 0.75		
					T/A 19 (31.15)			
*MGLL*	rs893294	121,143T/A	Intron 8	61	A/A 6 (9.84)	A: 0.25	0.54 (A)	0.75

^1 ^All SNPs were tested for Hardy-Weinberg equilibrium (exact p ≥ 0.05); ^2 ^The TGC haplotype comprises the minor alleles of rs806379, rs1535255, and rs2023239; ^3 ^Numbers are given according to genomic entry AL136096.7 and the translation start codon (nt+1 is the A of ATG); SNP alleles correspond to dbSNP ; ^4 ^Number of anorexia nervosa trios genotyped; ^5 ^Genotype frequencies in the patients with AN; ^6 ^Allele frequencies in the patients with AN; ^7 ^Transmission rate of the minor alleles; ^8 ^for TDT

The *CNR1 *haplotype comprising the minor alleles of rs806379, rs1535255 and rs2023239 (TGC), previously found to be associated with polysubstance abuse in European and African Americans [32], revealed likewise no evidence for an association with AN in our study groups. The allele frequencies of the *CNR1 *AAT trinucleotide repeat were in accordance to previous results [29,37,38]. However, the AAT_13 _and AAT_14 _repeat alleles, previously found to be preferentially transmitted to the restricting and binge eating/purging type of AN, respectively, [29] did not indicate evidence for an association with AN in our samples (the global test for transmission disequilibrium indicated p = 0.35). Further haplotype analyses resulted in lack of transmission disequilibrium for all haplotypes, including those solely comprising frequently transmitted alleles (p of global test for haplotypes of five *CNR1 *SNPs: 0.66; five *FAAH *SNPs: 0.45 and four *NAAA *SNPs: 0.72).

## Discussion

We observed no evidence for a transmission disequilibrium for any of the 15 analysed SNPs in *CNR1*, *FAAH, NAAA or MGLL *as assessed by the TDT. The strongest effect with an estimated transmission rate of 59% hinting at a preferential transmission of the *CNR1 *rs1049353 A-allele to patients with AN was not substantiated in a subsequent case-control study comprising 113 independent patients with AN and 178 healthy controls. Also, combining the 113 independent patients with AN with the 91 patients from study group 1 did not alter this lack of evidence for an association of the rs1049353 A-allele to patients with AN. Contrary to our initial expectation of an effect size of 1.43 estimated from the trio sample, the true effect of this allele may be more moderate to be detected by our, even pooled, relatively small case-control sample.

Even though the *CNR1 *SNP rs1049353 has not been analysed in patients with AN before, the G-allele of rs1049353 was recently found to be associated with obesity in a small case-control study comprising obese and normal weight Italians [39]. However, several other studies could not confirm this finding [35,40,41]. The *CNR1 *TGC haplotype previously found to be associated with polysubstance abuse in European and African Americans [32] revealed likewise no evidence for a transmission disequilibrium in our samples. Also the AAT_14 _and AAT_13 _repeat alleles of *CNR1*, which has previously been reported to be preferentially transmitted in patients with the binge eating/purging or restricting type of AN, respectively, were not found to be preferentially transmitted to patients with AN in our study. However, it has to be considered that the moderate to low sample size used in this study might have contributed to the observed lack of association. Additionally, it can not be ruled out that other variants, that are not in linkage disequilibrium with the here analysed variants, might contribute to the pathogenesis of AN.

Exogenous and endogenous cannabinoids stimulate food intake and promote weight gain in both, rodents [42] and humans [12,13]. Oral application of Δ^9^-THC further increases food intake and entails weight restoration in cachectic patients receiving cancer chemotherapy [43,44]. Only one small trial comprising 11 (of which three dropped out) patients with AN has focused on body weight gain after treatment with Δ^9^-THC and found no effect of oral application of Δ^9^-THC in doses up to 30 mg/day on body weight gain after four weeks of treatment [45]. However, in light of the small sample size, this observation has to be regarded with caution, especially as THC induced weight gain was compared to diazepam, for which animal studies also indicate a stimulation of body weight gain after its application [46,47].

Various association studies for AN have, with only limited success, focused on genes implicated in the regulation of food intake, as e.g. on the genes coding for leptin [48], the leptin receptor (*ObRb*) [49], ghrelin [50,51], the brain derived neurotrophic factor (*BDNF*) [52-55] or the tumor necrosis factor-alpha (*TNF-α*) [56]. Most of the respective studies yielded negative results. However, Ribases et al. (2003) reported strong association of the Val66Met polymorphism of *BDNF *with the restricting type of AN [52]. Additionally, this variant was associated with minimum BMI in these patients [52]. Replication analyses of this variant in 1,142 Caucasian patients with eating disorders from five European countries confirmed the association of this variant with all eating disorder subtypes including AN, AN-restricting type, AN-binge-eating/purging type and BN [53]. However, not all studies were able to confirm this finding [55].

## Conclusion

In summary, we did not find evidence for an association of the (AAT)_n _repeat and several SNPs in *CNR1*, *FAAH, NAAA and MGLL *with AN in our samples. We thus conclude that the here analysed variations in *CNR1, FAAH, NAAA *and *MGLL *at least do not seem to play a major role in the genetic etiology of AN in our study groups.

## Competing interests

The authors declare that they have no competing interests.

## Authors' contributions

TDM carried out the allele specific PCR, participated in design and interpretation of data and drafted the manuscript. KR and JK carried out the capillary sequencing. KR further participated in design and interpretation of data and revised the manuscript critically. GB participated in the design and interpretation of data. TTN and AS performed the statistical analysis under supervision of HS. WH and BH-D participated in patient recruitment and interpretation of data. PL and TM carried out the molecular genetic studies using MALDI-TOF mass spectrometry. JH and AH conceived the design and participated in coordination and interpretation of data; helped to draft the manuscript and revised it critically.
